# Characterization of Escherichia Phage ND-4 Against Avian Pathogenic *Escherichia coli* IMT5155 and Its Preliminary Application in Chicks

**DOI:** 10.3390/life16091549

**Published:** 2026-09-16

**Authors:** Leping Wang, Jiahe Zhou, Guotai Liu, Qinting Dong, Huili Bai, Changting Li, Yongcui Feng, Dongyan Deng, Yangyan Yin, Chunxia Ma, Ling Teng, Honggan Wei, Ruofu Qin, Xian Li, Yanwen Zhang, Hao Peng

**Affiliations:** 1 Key Laboratory of China (Guangxi)-ASEAN Transboundary Animal Disease Prevention and Control, Ministry of Agriculture and Rural Affairs, Nanning 530001, China; wangleping96@foxmail.com (L.W.); baihuili2020@163.com (H.B.); lctyq0508@163.com (C.L.); kkdfyc@163.com (Y.F.); d1911245648@163.com (D.D.); 151779061414@163.com (Y.Y.); machunxia1213@163.com (C.M.); 15208987486@163.com (L.T.); 13878168951@163.com (R.Q.); 2 Guangxi Key Laboratory of Veterinary Biotechnology, Guangxi Veterinary Research Institute, Nanning 530001, China; 3 School of the Integrated Traditional Chinese and Western Medicine, Hunan University of Chinese Medicine, Changsha 410208, China; zjiahe333@163.com; 4 College of Animal Science and Technology, Guangxi University, Nanning 530004, China; liuguotai2001@163.com; 5 College of Animal Science and Technology, Guangxi Agricultural Vocational University, Nanning 530007, China; dongqinting@foxmail.com; 6 College of Animal Science and Technology, Guangxi Agricultural Engineering Vocational Technical College, Chongzuo 530028, China; 13517801758@163.com (H.W.); gxngydky@163.com (X.L.); 7 Nanning University, Nanning 530299, China

**Keywords:** *Escherichia coli*, Bacteriophage, APEC, genomic analysis

## Abstract

Colibacillosis caused by avian pathogenic *Escherichia coli* (APEC) remains an important bacterial disease in poultry production and is increasingly difficult to control because of antimicrobial resistance. In this study, a lytic phage, ND-4, was isolated from poultry-farm sewage using the O2:K1 APEC strain IMT5155 as the host, and its biological characteristics, genome features and preliminary application potential were evaluated. ND-4 formed clear plaques on IMT5155 and showed a tailed morphology, with an elliptical head of 110 ± 5 nm by 82 ± 5 nm and a tail of 97 ± 5 nm by 18 ± 2 nm, giving a total virion length of approximately 204 ± 10 nm. The phage had an optimal multiplicity of infection of 0.01, a latent period of 20 min, a 50 min burst period and a burst size of 231 PFU/cell. ND-4 remained active across pH 4–9 and at temperatures up to 60 °C, and it retained titers above 10^8^ PFU/mL after exposure to chloroform concentrations up to 15%. Whole-genome sequencing revealed a 154,669 bp double-stranded DNA genome with 184 predicted open reading frames and a G + C content of 48.98%. No virulence or antimicrobial resistance genes were detected. ND-4 reduced biofilm biomass in vitro and decreased tissue bacterial loads and alleviated hepatic inflammation and vacuolar degeneration in IMT5155-challenged chicks. These results indicate that ND-4 is a promising candidate for further development as a phage-based control agent against APEC infection, although larger in vivo studies are needed to define its efficacy, dosing window and biosafety profile.

## 1. Introduction

*Escherichia coli* (*E. coli*) is a Gram-negative bacterium in the family Enterobacteriaceae. It mainly colonizes the lower intestine of warm-blooded animals and is released into the environment through fecal shedding [1]. Although many *E. coli* strains are commensal, pathogenic strains can cause diarrhea and systemic disease in humans and animals. Before molecular virulence typing became widely used, *E. coli* strains were classified primarily by the O (lipopolysaccharide), H (flagellar), and K (capsular) antigens. Approximately 186 O groups and 53 H types have been described [2,3].

Avian pathogenic *Escherichia coli* (APEC) is an important cause of economic losses in the poultry industry. Continued studies of APEC have shown that some virulence factors in avian and livestock strains are also associated with extraintestinal infections in mammals [4,5]. APEC and human extraintestinal pathogenic *E. coli* (ExPEC), including O1, O2, and O18 serotypes, are phylogenetically related and share several genetic and pathogenic features. IMT5155 belongs to serotype O2:K1:H5, sequence type complex ST95, and sequence type ST140. It is closely related to ST95 APEC O1:K1 and human ExPEC O18:K1 strains [6,7]. These genomic similarities, together with the relatively weak host restriction of some *E. coli* lineages, raise concerns about cross-host transmission and shared pathogenic mechanisms [8].

Antibiotics, especially quinolones and ciprofloxacin, have been widely used to treat *E. coli* infection [9]. However, extensive antibiotic use has contributed to the emergence and spread of antimicrobial-resistant *E. coli*, creating an urgent need for alternative control strategies. Lytic bacteriophages are attractive candidates because of their host specificity and their potential to target pathogenic bacteria without broadly disrupting commensal microbiota.

Bacteriophages are abundant viruses that specifically infect bacteria [10]. Unlike conventional antibiotics, lytic phages can adsorb to host cells, replicate, and release progeny phages through bacterial lysis. Their host specificity may help preserve normal microbial communities in humans and animals [11]. Previous studies have shown that phages can be used to control bacterial infections and may be combined with antibiotics to enhance antibacterial activity [12,13,14,15]. Nevertheless, clinical and agricultural applications remain limited, partly because bacterial resistance can also evolve against phages. Continued isolation and characterization of *E. coli* phages therefore remain important for developing multi-phage preparations.

In this study, we isolated phage ND-4 from farm sewage using the APEC strain IMT5155 as the host. We evaluated its host range, biological characteristics, genome content, biofilm-clearance activity, chloroform tolerance, and preliminary therapeutic effect in chicks. These data provide a basis for further development of phage-based agents against multidrug-resistant *E. coli*.

## 2. Materials and Methods

### 2.1. Materials

#### 2.1.1. Time and Location

The experiments were completed in September 2025 at the Guangxi Zhuang Autonomous Region Veterinary Research Institute.

#### 2.1.2. Bacterial Strains

This study used 40 *E. coli* strains and 2 *Salmonella* strains. Twenty-two *E. coli* strains were provided by the Clinical Diagnosis Laboratory, College of Animal Science and Technology, Guangxi University. Fifteen *E. coli* strains were isolated from a farm in Guangxi and provided by the Bacteriology Laboratory of the Guangxi Veterinary Research Institute. Two *E. coli* strains and two *Salmonella* strains were purchased from the China Veterinary Culture Collection Center. The host strain, *E. coli* IMT5155 (APEC), was provided by Researcher Shaohui Wang of the Shanghai Veterinary Research Institute.

#### 2.1.3. Main Reagents

The main reagents included 0.22 μm sterile filters (Biosharp, Beijing, China), LB broth and LB agar medium (Beijing Luqiao Technology Co., Ltd., Beijing, China), agar powder (Solarbio Science & Technology Co., Ltd., Beijing, China), a viral nucleic acid extraction kit, DNase, and RNase A (CoWin Biosciences, Taizhou, China).

### 2.2. Methods

#### 2.2.1. Isolation, Purification, and Morphological Observation

Sewage samples were collected from a poultry farm in Nanning, Guangxi. Samples were centrifuged at 3000 r/min, and the supernatant was added to logarithmic-phase *E. coli* IMT5155 cultures (OD_600_ = 0.4–0.5). The mixture was incubated at 37 °C with shaking for 12–18 h. The culture was then filtered through a 0.22 μm sterile filter to obtain the initial phage suspension.

Phage isolation was performed using the double-layer agar method. Briefly, 100 μL of phage suspension and 100 μL of bacterial culture were mixed with LB semi-solid agar at approximately 50 °C, poured onto LB agar plates, and incubated at 28 °C for 12–18 h. Single plaques with regular morphology were picked into SM buffer and resuspended for 30 min. Purification was repeated three to five times until plaque morphology was uniform. The purified phage was negatively stained with phosphotungstic acid and examined by transmission electron microscopy at Chengdu Lilai Biotechnology Co., Ltd. (Chengdu, China)

#### 2.2.2. Host Range Determination

The host range of phage ND-4 was initially screened using a spot assay against 40 *E. coli* strains and two *Salmonella* strains. Strains showing clear lysis zones were further evaluated using the double-layer agar plaque assay. Phage titers were determined for each susceptible strain, and the efficiency of plating (EOP) was calculated as the ratio of the phage titer obtained on the test strain to that obtained on the original host strain, *E. coli* IMT5155. All assays were performed in triplicate.

$$EOP=\frac{Phage\;titer\;on\;test\;strain}{Phage\;titer\;on\;host\;strain\;IMT5155}$$

#### 2.2.3. Determination of Biological Characteristics

Optimal MOI was determined by mixing *E. coli* IMT5155 (1 × 10^6^ CFU/mL) with phage at ratios of 100, 10, 1, 0.1, and 0.01. The mixtures were incubated at 37 °C and 150 r/min for 6 h. Phage titers were then measured using the double-layer agar method, and the MOI producing the highest titer was selected. Each assay was performed in triplicate.

For pH stability assays, phage suspension (10^9^ PFU/mL) was added to SM buffer adjusted to pH 1.0–12.0. After incubation at 37 °C for 60 min, phage titers were measured.

For one-step growth analysis, host bacteria and phage were mixed at the optimal MOI of 0.01 and incubated at 37 °C. Samples were collected every 10 min for 180 min to measure phage titers. The latent period, burst period, and burst size were then calculated.

For thermal stability assays, phage suspensions (10^9^ PFU/mL) were incubated at 4, 25, 37, 50, 60, 70, or 80 °C for 30, 60, or 90 min. Samples were cooled in ice water for 2 min before titer determination.

#### 2.2.4. Whole Genome Sequencing and Analysis

Viral nucleic acid was extracted using the CoWin kit and verified by 1.2% agarose gel electrophoresis. Sequencing was performed by Shanghai Personalbio (Personalbio Technology Co., Ltd., Shanghai, China) on the Illumina NovaSeq 6000 platform. Raw reads were quality-controlled using SOAPnuke (v2.1.5), and host-derived sequences were removed using BWA (v0.7.17). Genome assembly was performed using MEGA HIT (v1.1.2), and contigs were evaluated using CheckV (v1.0.3).

Open reading frames were predicted using the RAST online platform (https://rast.nmpdr.org/, accessed on 23 June 2025) and annotated against the COG database. Virulence and antimicrobial resistance genes were screened using RAST and PHASTER. A phylogenetic tree based on the major capsid protein was constructed in MEGA 11 using the neighbor-joining method. Genome synteny was visualized using Easyfig 2.2.3.

#### 2.2.5. Preliminary Application Experiments

For chloroform sensitivity assays, phage suspensions (10^9^ PFU/mL) were treated with chloroform at final concentrations of 1%, 3%, 5%, 10%, and 15%. The mixtures were incubated at 37 °C and 170 r/min for 30 min, centrifuged at 11,000 r/min and 4 °C for 1 min, and the supernatants were collected for titer determination.

For biofilm inhibition assays, bacterial suspension (1 × 10^9^ CFU/mL) was added to 24-well plates at 500 μL per well and incubated at 37 °C for 72 h to allow biofilm formation. Wells were washed with PBS, and phage suspensions were added at MOIs of 1, 0.1, and 0.01. After 12 h of incubation, wells were washed, fixed with methanol for 15 min, stained with crystal violet for 15 min, and solubilized with methanol. Biofilm biomass was assessed by measuring OD_590_.

#### 2.2.6. Chick Infection and Treatment Assay

Before the animal experiment, the experimental site was fumigated with potassium permanganate and formaldehyde at a 1:2 ratio and then fully ventilated. Commercially sourced fertilized specific-pathogen-free chicken eggs were used. Newly hatched chicks were given a multivitamin and glucose solution before 3 days of age. The chicks were randomly divided into four experimental groups: the negative control group (*n* = 20), positive control group (*n* = 20), antibiotic-treatment group (*n* = 20), and phage-treatment group (*n* = 20).

On day 4, each chick in the three infection groups was intraperitoneally inoculated with 20 μL of IMT5155 suspension, corresponding to approximately 5 × 10^7^ CFU. The negative control group received no IMT5155 challenge. After infection, the chicks were transferred to cages that met animal welfare requirements and were provided with autoclaved double-distilled water and high-temperature-sterilized starter feed ad libitum.

Treatment was initiated 3 days after challenge, at 7 days of age. The antibiotic-treatment group received gentamicin soluble powder in the drinking water at the recommended dose, whereas the phage-treatment group received ND-4 at approximately 1 × 10^8^ PFU/mL in the drinking water. The average daily water consumption was approximately 20 mL per chick. Both treatments were administered continuously for 3 days. No additional antibiotics or phages were administered after completion of the 3-day treatment period.

Chick health status was evaluated twice daily throughout the experimental period. Body weight was recorded at 10 and 14 days of age. At the end of the experiment, the surviving chicks were euthanized by carotid artery bleeding. Liver and spleen tissues with major lesions were collected and divided into two portions. One portion was placed in 1000 μL sterile PBS and mechanically homogenized with grinding beads for bacterial-load determination. The other portion was fixed in 4% paraformaldehyde for 48 h, dehydrated, embedded, sectioned, and stained with hematoxylin and eosin for histopathological analysis.

The animal experiment was approved by the Animal Ethics Committee of Guangxi Veterinary Research Institute (Approval No. GX-2025-033).

#### 2.2.7. Statistical Analysis

Data are presented as the mean ± standard deviation (SD). Statistical analyses were performed using GraphPad Prism 9.0. Survival curves were generated using the Kaplan–Meier method and compared using the Log-rank (Mantel–Cox) test. Longitudinal body-weight data were analyzed using a mixed-effects model, with treatment group and time as fixed effects, followed by Tukey’s multiple-comparison test. This approach was used because mortality resulted in missing body-weight measurements at later time points. Bacterial loads in the spleen and liver were analyzed separately using one-way ANOVA followed by Tukey’s multiple-comparison test. For other datasets involving comparisons among multiple independent groups, one-way ANOVA followed by Tukey’s multiple-comparison test was used. A value of *p* < 0.05 was considered statistically significant.

## 3. Results

### 3.1. Isolation and Morphology

**Figure 1:**
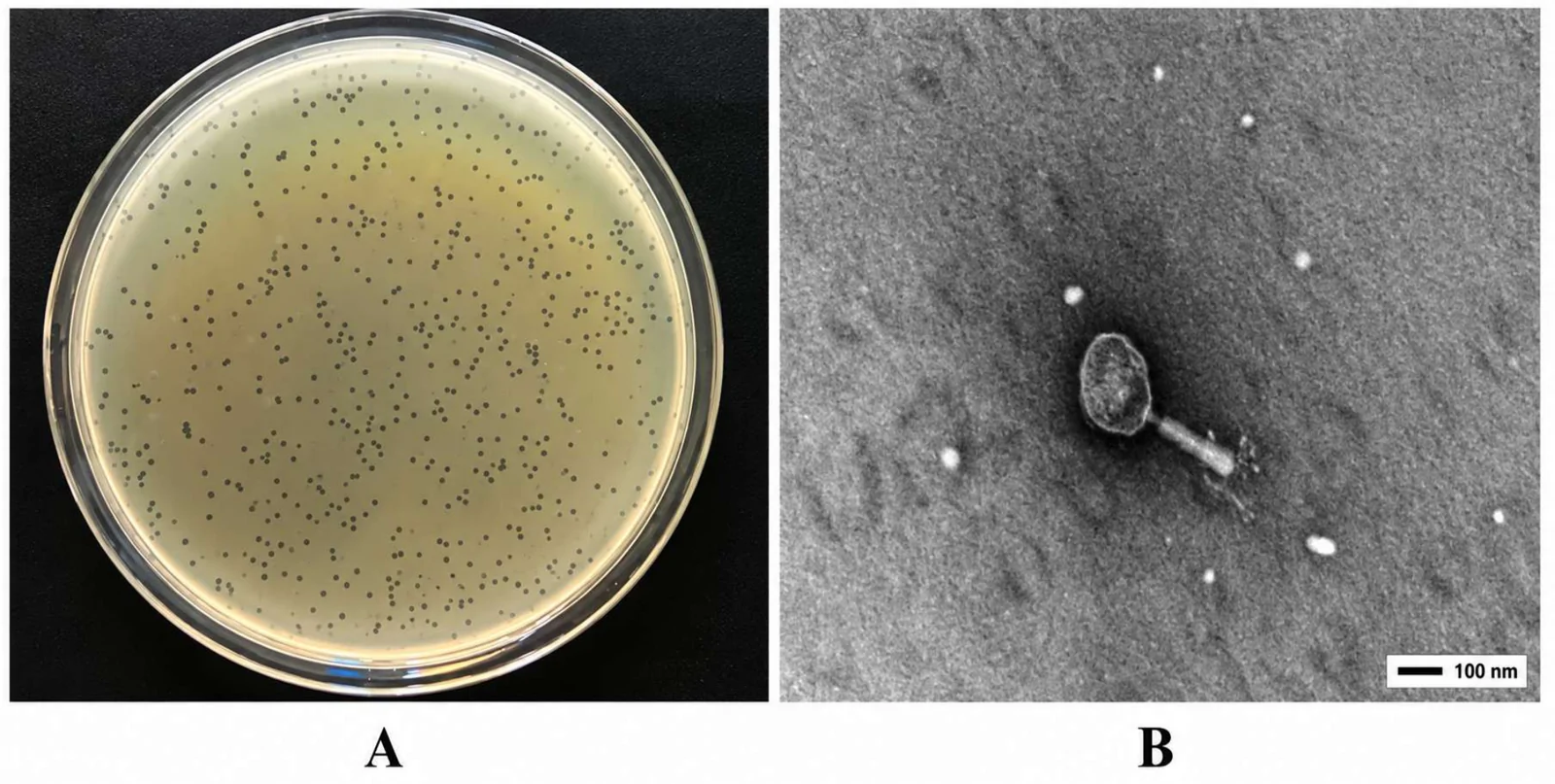
Plaque morphology and virion structure of ND-4. (**A**) Plaque morphology. (**B**) Transmission electron microscopy image.

Transmission electron microscopy showed that ND-4 possessed an elliptical head measuring 110 ± 5 nm (long axis) by 82 ± 5 nm (short axis) and a tail measuring approximately 97 ± 5 nm in length and 18 ± 2 nm in width (Figure 1A). Re-examination of the TEM images indicated that the tail morphology is consistent with a contractile tail rather than a non-contractile tail (Figure 1B). Together with the whole-genome and phylogenetic analyses, these morphological features support the assignment of ND-4 to the family *Ackermannviridae* within the class *Caudoviricetes*.

### 3.2. Host Range

ND-4 produced plaques on three of the 40 tested *E. coli* strains, including the original host IMT5155, CVCC1527, and D-GX3, whereas no plaques were detected on the remaining *E. coli* strains or the two *Salmonella* strains. The EOP on IMT5155 was defined as 1.0, while the EOP values on CVCC1527 and D-GX3 were 0.47 and 0.85, respectively (Table 1). These results indicate that ND-4 has a relatively narrow productive host range under the tested conditions.

### 3.3. Biological Characteristics

**Figure 2:**
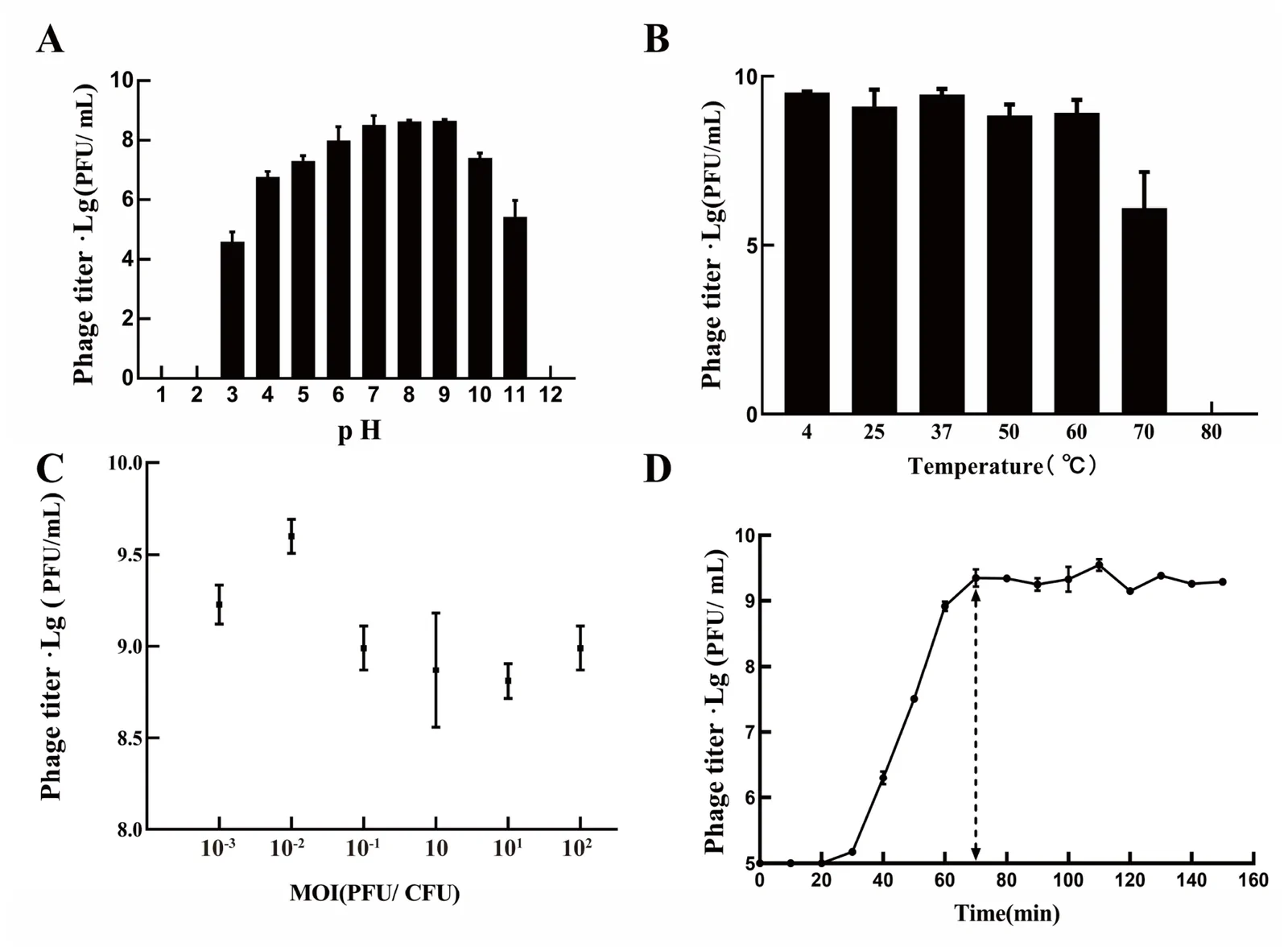
Biological characteristics of phage ND-4. (**A**) pH stability. (**B**) Thermal stability. (**C**) Optimal MOI determination. (**D**) One-step growth curve.

Thermal stability assays showed that ND-4 titers remained stable from 4 °C to 50 °C. At 60 °C, titers began to decline after 30 min. At 80 °C, activity persisted for 30 min but was lost by 60 min (Figure 2A).

ND-4 remained active from pH 3 to pH 11, with the highest titers observed at pH 7–9 (approximately 10^9^ PFU/mL). Activity was lost at pH 2 and pH 12 (Figure 2B).

One-step growth analysis showed a latent period of 20 min, followed by a 50 min burst period. The phage titer reached a plateau at approximately 70 min, with an estimated burst size of approximately 231 PFU/cell (Figure 2C).

The highest phage titer, 3.97 × 10^9^ PFU/mL, was obtained at an MOI of 0.01 after 6 h of incubation (Figure 2D).

### 3.4. Genomic Analysis

**Figure 3:**
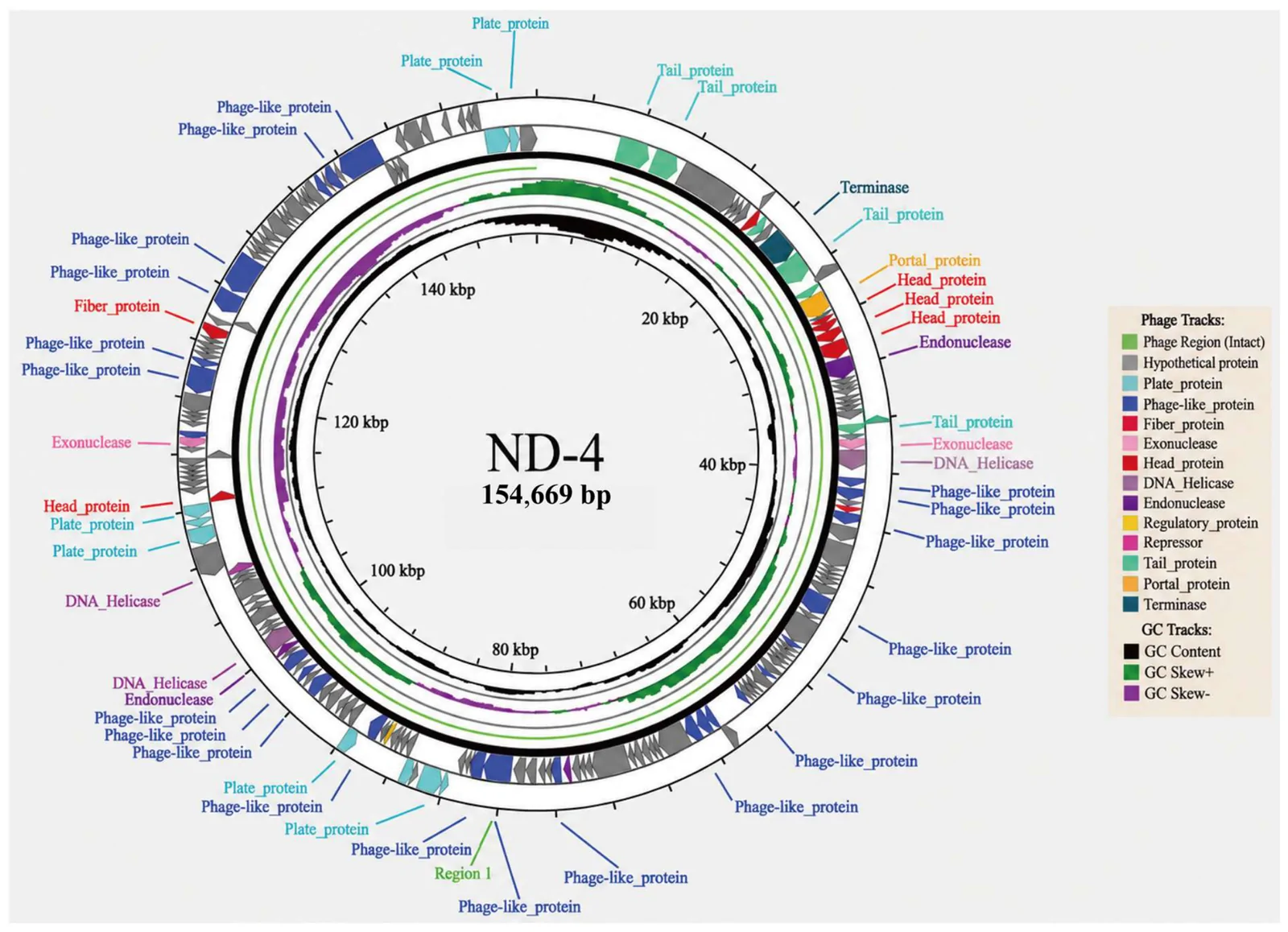
Genome map of phage ND-4.

The ND-4 genome is a double-stranded DNA genome of 154,669 bp (GenBank accession: PQ374922), with a G + C content of 48.98%. Genome annotation predicted 184 proteins. Forty-five proteins had assigned functions related mainly to structure, replication, or lysis, whereas the remaining proteins were annotated as hypothetical proteins. No virulence or antimicrobial resistance genes were detected (Figure 3 and Table 2).

**Figure 4:**
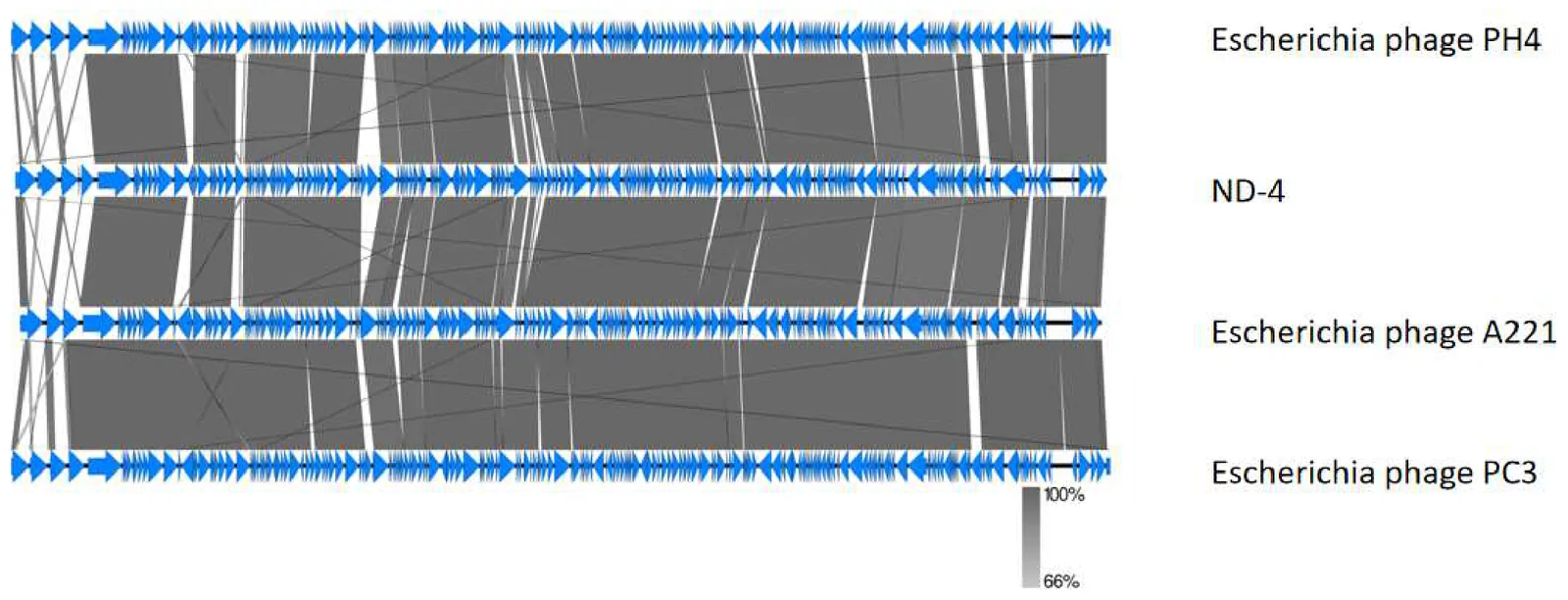
Genomic synteny analysis of ND-4 and related phages.

Functional annotation identified terminase, DNA helicase, regulatory proteins, portal protein, tail fiber proteins, capsid proteins, and lysis-related proteins, including lysozyme and endolysin. BLAST (https://blast.ncbi.nlm.nih.gov/, accessed on 23 June 2025) analysis showed that ND-4 was homologous to *Escherichia* phages A221, PC3, and PH4, with more than 98% nucleotide identity and 86–87% genome coverage (Figure 4).

**Figure 5:**
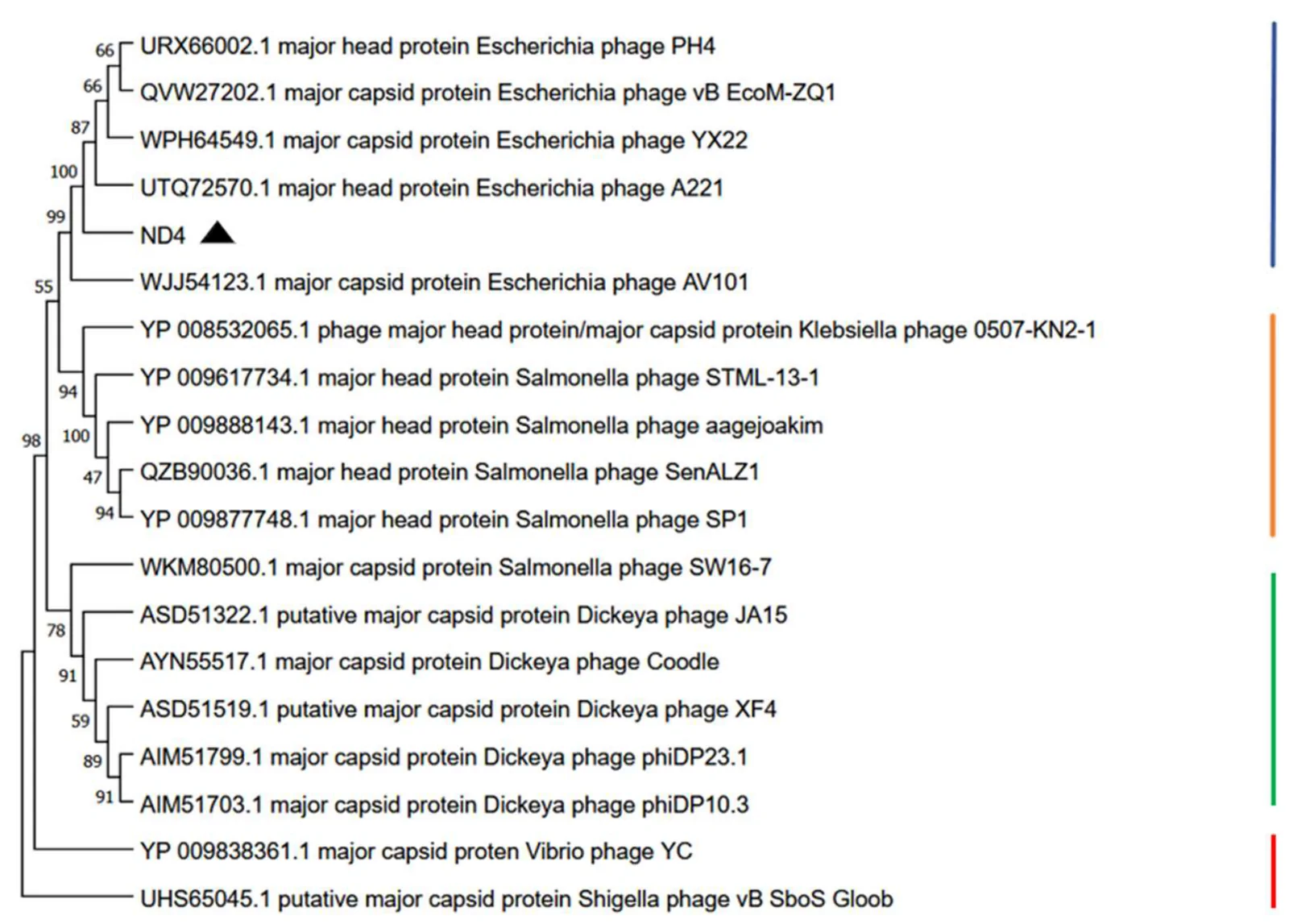
Phylogenetic tree based on the major capsid protein. Note: The triangle indicates the bacteriophage used in this study.

Phylogenetic analysis based on the major capsid protein (ORF22) clustered ND-4 with *Escherichia* phages A221, YX22, vB_EcoM-ZQ1, and PH44. Together with whole-genome similarity analysis, these phylogenetic relationships supported the assignment of ND-4 to the family *Ackermannviridae*, subfamily *Aglimvirinae*, within the class *Caudoviricetes*, in accordance with the current ICTV genomic classification framework (Figure 5). Re-examination of the TEM images further indicated that the tail morphology of ND-4 is consistent with a contractile-tail morphology, supporting its genomic assignment to the family *Ackermannviridae*.

### 3.5. Preliminary Application

**Figure 6:**
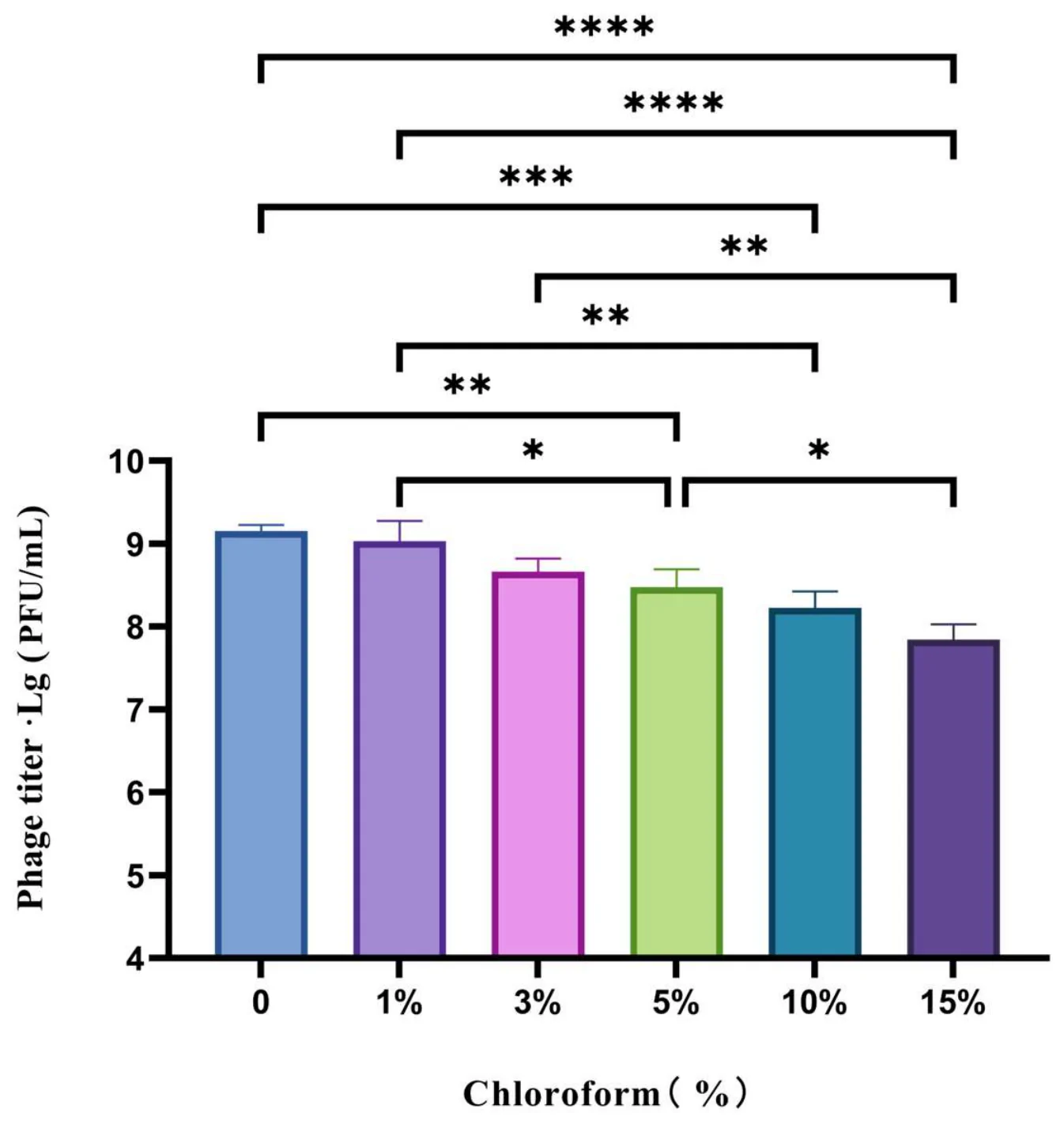
Chloroform sensitivity of phage ND-4. (* *p* < 0.05, ** *p* < 0.01, *** *p* < 0.001, and **** *p* < 0.0001 (one-way ANOVA followed by Tukey’s multiple-comparison test.)).

ND-4 maintained titers above 10^8^ PFU/mL after treatment with chloroform concentrations up to 15% (Figure 6). This result is consistent with ND-4 being a non-enveloped phage.

**Figure 7:**
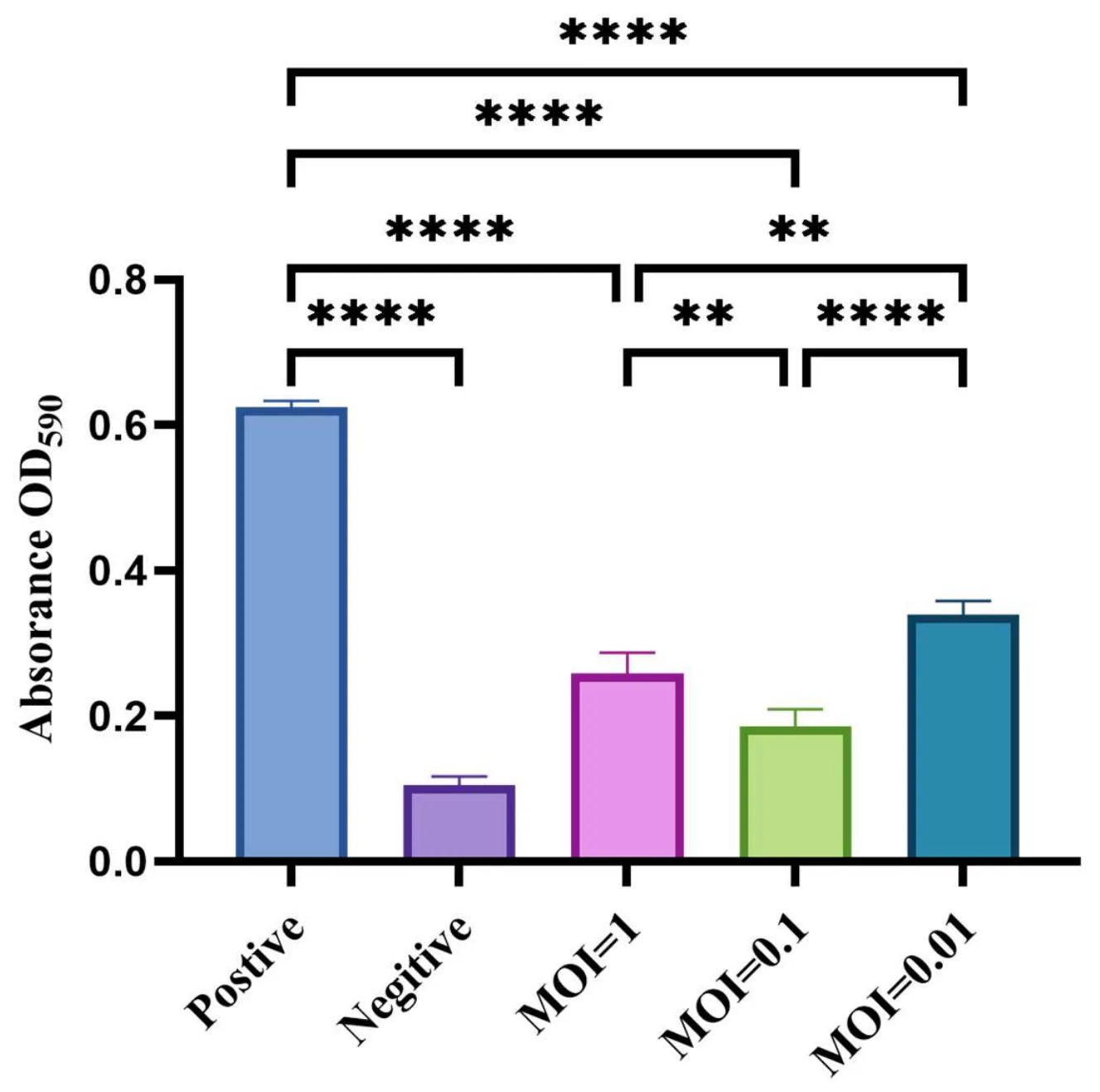
Biofilm-clearance efficiency of phage ND-4 at different MOIs. (** *p* < 0.01, and **** *p* < 0.0001 (one-way ANOVA followed by Tukey’s multiple-comparison test.)).

ND-4 significantly reduced biofilm biomass compared with the positive control (OD_590_ = 0.62) and approached the negative-control level (OD_590_ = 0.11). The strongest clearance effect was observed at an MOI of 0.1 (Figure 7).

### 3.6. Preliminary Chick Application Assay

**Figure 8:**
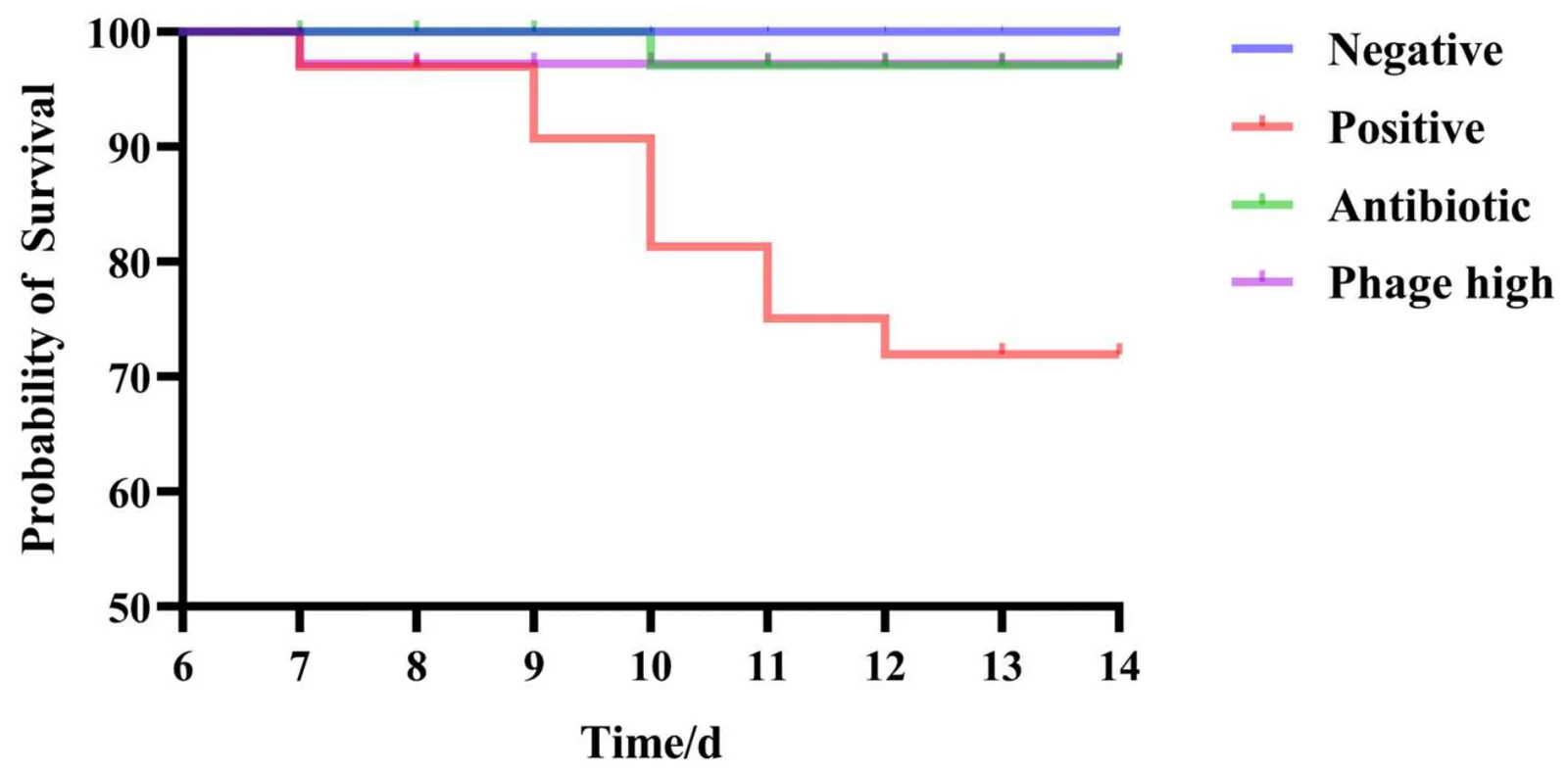
Survival curves of chicks (*n* = 20 per group).

Survival analysis showed no mortality in the negative control group during the rearing period (Figure 8). Before treatment, deaths occurred in all three IMT5155-challenged groups. After treatment, mortality decreased in the antibiotic and phage treatment groups.

**Figure 9:**
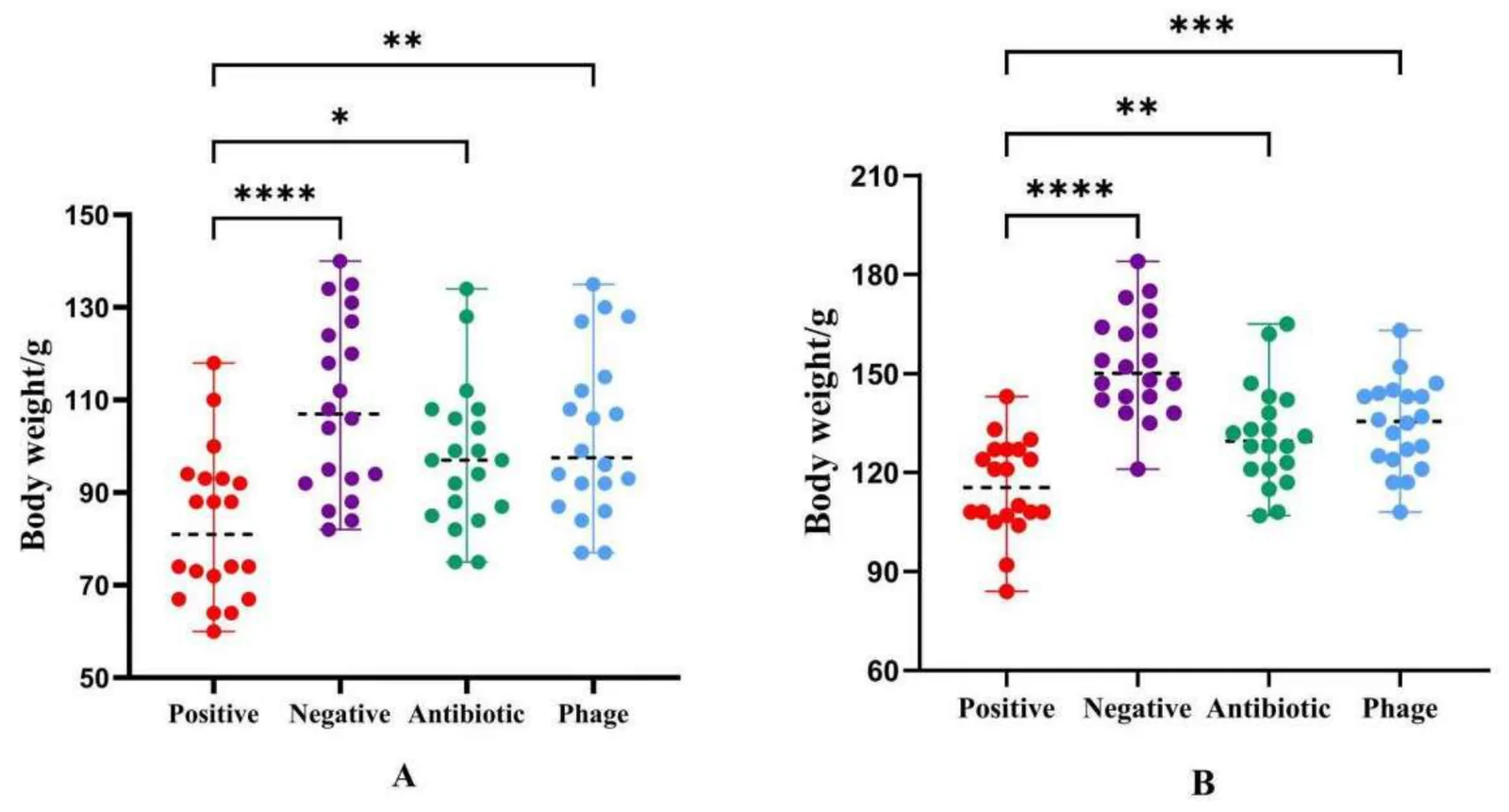
Body weight of chicks at 10 and 14 days of age. (**A**) Body weight at 10 days. (**B**) Body weight at 14 days. *(n* = 20 per group, * *p* < 0.05, ** *p* < 0.01, *** *p* < 0.001, and **** *p* < 0.0001 (one-way ANOVA followed by Tukey’s multiple-comparison test)).

Body-weight analysis showed that IMT5155 infection suppressed weight gain during the monitored periods (Figure 9). Chicks treated with ND-4 or antibiotics showed partial recovery of body weight.

**Figure 10:**
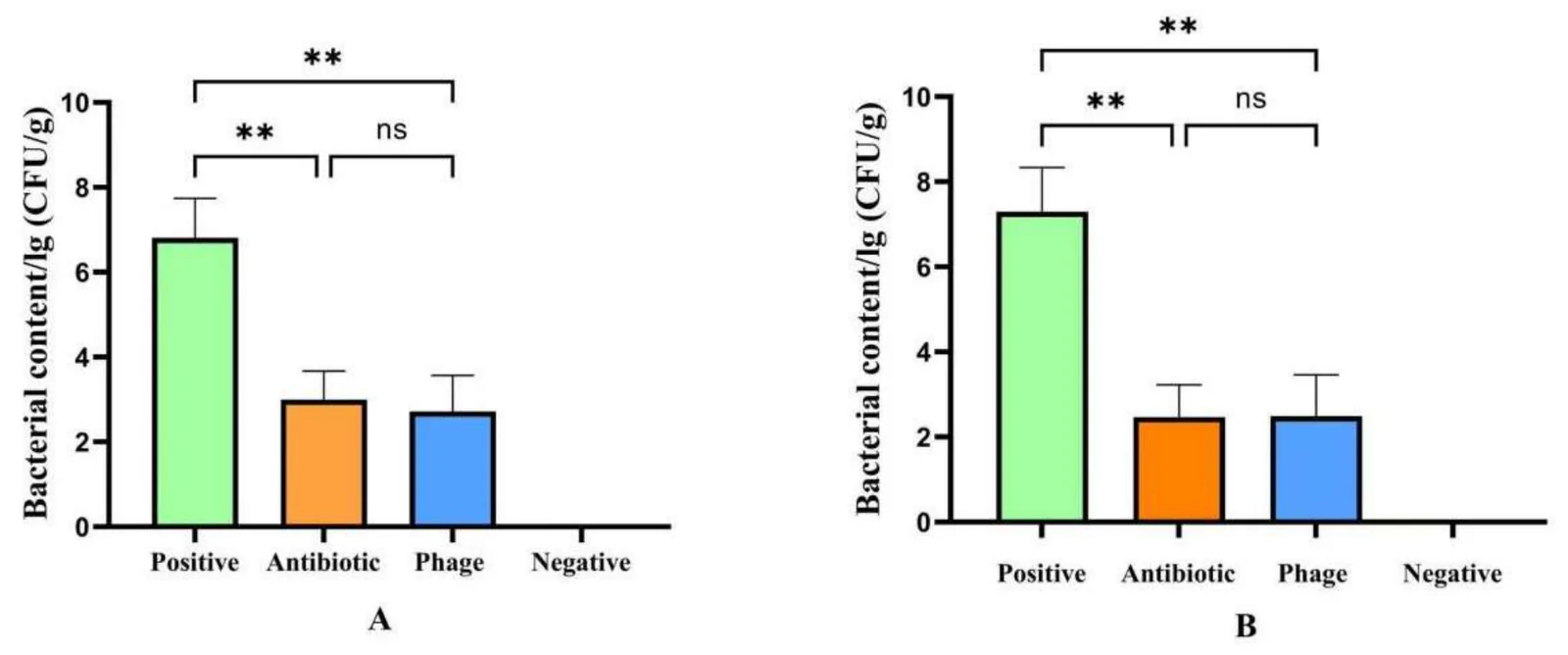
Bacterial load in chick tissues. (**A**) Bacterial load in spleen tissue. (**B**) Bacterial load in liver tissue. (** *p* < 0.01, ns = not significant, (one-way ANOVA followed by Tukey’s multiple-comparison test)).

Bacterial-load analysis showed that phage and antibiotic treatment markedly reduced bacterial loads in chick tissues compared with the IMT5155-infected positive control (Figure 10). The reduction was approximately four orders of magnitude.

**Figure 11:**
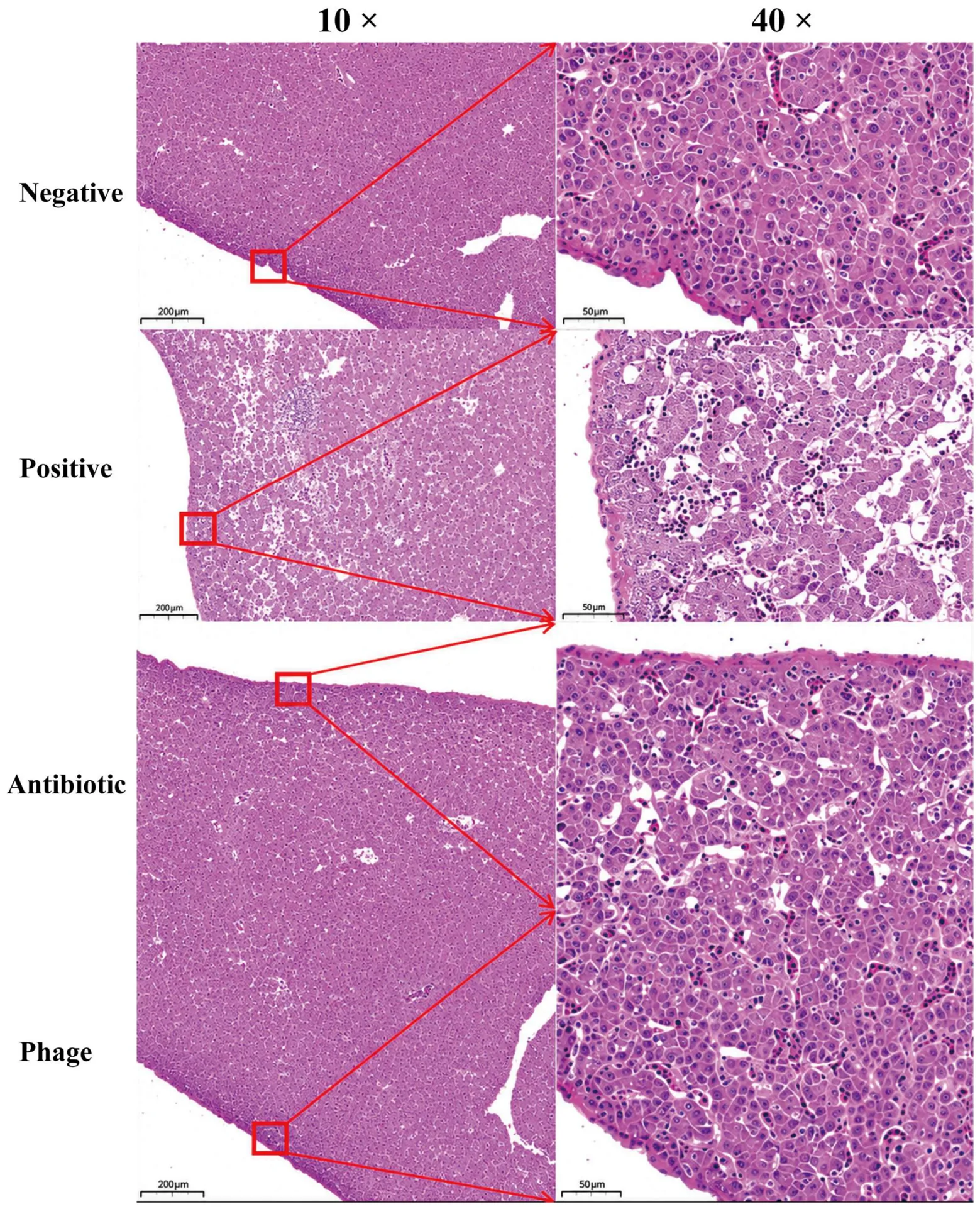
Histological sections of chick liver tissue.

Histopathological analysis showed marked hepatic interstitial congestion and vacuolar degeneration in IMT5155-infected chicks compared with the blank control group (Figure 11). Treatment with ND-4 or antibiotic reduced hepatic inflammation and steatosis. The phage-treated group showed slightly greater histological improvement than the antibiotic-treated group under the conditions tested.

## 4. Discussion

This study identified ND-4 as a lytic *E. coli* phage with favorable biological stability, genomic safety features and preliminary application potential against APEC IMT5155. The phage showed a narrow host range, lysing IMT5155, CVCC1527 and D3 but not the other tested *E. coli* or *Salmonella* strains. This specificity may be advantageous for targeted control, although it also indicates that ND-4 would probably need to be combined with complementary phages for broader field application.

The replication and stability profiles of ND-4 support its further evaluation as an applied phage candidate. ND-4 had a latent period of 20 min and a burst size of 231 PFU/cell, indicating efficient propagation on IMT5155. Its burst size was higher than that reported for phage A221 [16], and its environmental tolerance was also favorable, with activity across pH 3–11 and short-term retention of activity at 80 °C. These features are relevant for preparation, storage and delivery, although formulation strategies such as microencapsulation may still be needed to improve stability under practical farm conditions [17].

Genome analysis further supported the lytic and safety profile of ND-4. The genome contained 184 predicted open reading frames, including genes associated with structure, replication and lysis. Tail fiber proteins are likely to contribute to the observed host specificity, as receptor-binding proteins are key determinants of phage host range [18,19,20]. No integrase, virulence gene or antimicrobial resistance gene was detected, which is an important prerequisite for therapeutic or preventive development [21,22,23,24,25]. The predicted nicotinamide synthesis-related protein may also be relevant to phage-bacteria interactions because some phages use NAD-related strategies to counter bacterial defense systems [25,26].

ND-4 also showed activity in application-oriented assays. Chloroform tolerance was consistent with the non-enveloped nature of the phage, and biofilm assays showed that ND-4 reduced biofilm biomass at all tested MOIs. The strongest effect occurred at an MOI of 0.1 rather than at the highest tested phage concentration. This pattern suggests that biofilm clearance may depend not only on phage dose but also on phage diffusion, adsorption and movement within the biofilm matrix [27].

The chick infection assay provided preliminary evidence that ND-4 can reduce the pathological impact of IMT5155 challenge. Phage treatment improved survival, body weight, tissue bacterial load and liver histopathology compared with infected controls, and some outcomes were comparable to or slightly better than gentamicin treatment. These effects are consistent with the targeted antibacterial activity expected for lytic phages. Nevertheless, the animal data should be interpreted cautiously because dose–response effects, treatment timing, route of administration, microbiota changes and resistance emergence were not fully resolved.

Although ND-4 was administered orally through drinking water, whereas the IMT5155 challenge was performed intraperitoneally, previous studies suggest that orally administered bacteriophages can cross epithelial barriers and reach extraintestinal sites. Nguyen et al. demonstrated that diverse bacteriophages can undergo rapid apical-to-basolateral transcytosis across epithelial cell layers, providing a potential mechanism for phage entry into the systemic compartment after oral administration [28]. More directly, a recent study in laying hens showed that a phage cocktail administered through drinking water after systemic APEC challenge could be recovered from multiple internal tissues and significantly reduced bacterial-associated morbidity and pathological lesions, supporting the feasibility of oral phage delivery for controlling extraintestinal APEC infection [29]. Once phages reach infected tissues and encounter susceptible bacteria, local replication may further increase the effective phage concentration and enhance antibacterial activity, a phenomenon generally referred to as phage self-amplification [30]. These mechanisms may partly explain the therapeutic effects observed for ND-4 in the present study. However, because phage concentrations in blood and tissues were not directly measured, the systemic bioavailability, tissue distribution, and pharmacokinetics of orally administered ND-4 remain to be determined in future studies.

Overall, ND-4 expands the available phage resources for APEC control and provides a candidate for future cocktail design. Its narrow host range argues for combination with other well-characterized lytic phages, whereas its stability, lack of detectable risk genes, and preliminary in vivo efficacy support further development. Future studies should test ND-4 in larger animal cohorts, define optimal dosing regimens, evaluate resistance dynamics and assess performance in multi-phage preparations under conditions closer to poultry production.

## 5. Conclusions

ND-4 is a newly characterized lytic *E. coli* phage isolated against the APEC strain IMT5155. It showed efficient propagation, broad environmental tolerance, biofilm-reduction activity and preliminary protection in challenged chicks. Its genome lacked detectable virulence and antimicrobial resistance genes, supporting further safety assessment. These findings indicate that ND-4 is a promising candidate for phage-based APEC control, especially as part of a rationally designed cocktail, but its efficacy and biosafety require validation in larger and more practical infection models.

## Figures and Tables

**Table 1:** Host-range analysis of phage ND-4.

Strains	Genus	Source	Serotypes	Lytic
IMT5155	*E.coli*	Chicken	O2:K1	+
CVCC1527	*E.coli*	Pig	O8:K88	+
CVCC4050	*E.coli*	Pig	O157:H7	-
GXEC-01	*E.coli*	Chicken	NA	-
GXEC-02	*E.coli*	Chicken	NA	-
GXEC-03	*E.coli*	Chicken	O157	-
GXEC-04	*E.coli*	Chicken	NA	-
GXEC-05	*E.coli*	Chicken	NA	-
GXEC-06	*E.coli*	Chicken	O157	-
GXEC-07	*E.coli*	Chicken	NA	-
GXEC-08	*E.coli*	Chicken	NA	-
GXEC-09	*E.coli*	Chicken	NA	-
GXEC-10	*E.coli*	Chicken	NA	-
GXEC-11	*E.coli*	Chicken	O142:K68	-
GXEC-12	*E.coli*	Chicken	NA	-
GXEC-13	*E.coli*	Chicken	NA	-
GXEC-14	*E.coli*	Chicken	NA	-
GXEC-15	*E.coli*	Chicken	NA	-
HZDC01	*E.coli*	Chicken	NA	-
HZDC02	*E.coli*	Chicken	NA	-
HZDC03	*E.coli*	Chicken	O114:K90	-
HZDC04	*E.coli*	Chicken	NA	-
HZDC05	*E.coli*	Chicken	NA	-
HZDC06	*E.coli*	Chicken	NA	-
HZDC07	*E.coli*	Chicken	NA	-
HZDC08	*E.coli*	Chicken	O26:K60	-
HZDC09	*E.coli*	Chicken	NA	-
D-GX1	*E.coli*	Chicken	NA	-
D-GX2	*E.coli*	Chicken	NA	-
D-GX3	*E.coli*	Chicken	NA	+
D-GX4	*E.coli*	Chicken	NA	-
D-GX5	*E.coli*	Chicken	NA	-
D-GX6	*E.coli*	Chicken	NA	-
C-D1	*E.coli*	Chicken	O157:H7	-
C-D2	*E.coli*	Chicken	NA	-
C-D3	*E.coli*	Chicken	NA	-
C-D4	*E.coli*	Chicken	NA	-
C-D5	*E.coli*	Chicken	O2	-
C-D6	*E.coli*	Chicken	NA	-
C-D7	*E.coli*	Chicken	NA	-
C-D8	*E.coli*	Chicken	NA	-
CVCC1806	*Sal*	\	*S. enteritidis*	-
CVCC3384	*Sal*	\	*S. typhimurium*	-

Note: +, lytic activity; -, no lytic activity. Strains listed as serotype NA were tested using commercial O2, O26, O142, and O157 detection kits but were not assigned to any of these serotypes.

**Table 2:** Functional CDS annotation of phage ND-4.

CDS	Identity	Length (aa)	Function	e-Value	Accession Number
2	96.13%	3027	Phage tail fibers	0	WPH64569.1
3	82.03%	2430	Tail protein	0	YP_010659927.1
5	98.11%	1728	Tail spike protein	0	YP_010659925.1
8	100.00%	753	Putative head–tail protein	0	QZI78659.1
11	100.00%	696	tail stabilization protein	2 × 10^−173^	UTQ72558.1
13	90.32%	343	Terminase large subunit	0	UHHS65537.1
14	99.52%	1896	Tail sheath protein	0	UHS65542.1
17	100.00%	1695	Portal protein	7 × 10^−28^	URX65806.1
22	99.77%	1323	Major capsid protein	0	QVW27202.1
34	100.00%	453	Recombination mediator protein	6 × 10^−105^	WP_242243974.1
36	99.80%	1509	DNA helicase	0	WPH64536.1
39	100.00%	990	Sliding clamp loader subunit	0	WPH64532.1
40	100.00%	423	DNA polymerase	1 × 10^−97^	WP_276339478.1
41	100.00%	495	Translational repressor	4 × 10^−111^	WP_242244075.1
49	98.94%	855	Ribose-phosphate	0	WKM80470.1
50	99.64%	1665	Nicotinamide	0	WPH64521.1
65	100.00%	279	DNA-binding protein	7 × 10^−57^	WP_276339458.1
66	99.48%	1719	DNA helicase	0	UTQ72610.1
68	100.00%	528	RnaseH	1 × 10^−125^	QVW27164.1
71	99.61%	2346	Endonuclease subunit	0	UHS65534.1
84	100.00%	912	DNA primase	0	WPH64489.1
89	99.87%	2283	Ribonucleotide reductase	0	WPH64482.1
90	100.00%	1104	Ribonucleotide reductase	0	EJS6255053.1
91	100.00%	162	Glutaredoxin	3 × 10^−29^	WP_242244087.1
94	100.00%	381	GPW/gp25 family protein	6 × 10^−88^	EJS6255058.1
95	100.00%	1611	Lysozyme	0	UHS65547.1
105	99.41%	1023	ssDNA binding protein	0	WPH64466.1
119	99.79%	1425	DNA helicase	0	UHS65553.1
125	99.16%	1431	DNA ligase	0	WPH64446.1
128	99.54%	660	DNA helicase loader	7 × 10^−162^	QVW27103.1
147	100.00%	1332	DNA topoisomerase II medium subunit	0	USL85959.1
148	99.84%	1911	DNA topoisomerase	0	WPH64598.1
154	99.63%	810	tail protein	0	URX65943.1
173	99.50%	3000	DNA polymerase	0	USL85796.1

Note: Proteins not labeled in the table were annotated as hypothetical proteins with unknown function.

## Data Availability

The annotated complete genome sequence of the phage ND-4 was deposited in GenBank under accession number PQ374922.
